# Relationships between Patient-Reported Outcome Measures and Clinical Measures in Naïve Neovascular Age-Related Macular Degeneration Patients Treated with Intravitreal Ranibizumab

**DOI:** 10.3390/ph17020157

**Published:** 2024-01-25

**Authors:** Pablo Almuiña-Varela, Laura García-Quintanilla, María José Rodríguez-Cid, María Gil-Martínez, Maximino J. Abraldes, Francisco Gómez-Ulla, Ana Estany-Gestal, Jorge Miguel Alcántara-Espinosa, Maribel Fernández-Rodríguez, Anxo Fernández-Ferreiro

**Affiliations:** 1 Ophthalmology Department, University Clinical Hospital of Santiago de Compostela, (SERGAS), 15706 Santiago de Compostela, Spain; pablo.almuina.varela@sergas.es (P.A.-V.); maricardo@live.com (M.J.R.-C.); mariagilmtez@hotmail.com (M.G.-M.); maxiabraldes@gmail.com (M.J.A.); 2 Clinical Pharmacology Group, Health Research Institute of Santiago de Compostela (IDIS), 15706 Santiago de Compostela, Spain; lauragarqu@gmail.com; 3 Pharmacy Department, University Clinical Hospital of Santiago de Compostela (SERGAS), 15706 Santiago de Compostela, Spain; 4 Instituto Oftalmológico Gómez-Ulla, 15706 Santiago de Compostela, Spain; franciscogomez-ulla@institutogomez-ulla.es; 5 Department of Surgery, University of Santiago de Compostela, 15782 Santiago de Compostela, Spain; 6 FIDIS-Unidad de Epidemiología e Investigación Clínica, 15706 Santiago de Compostela, Spain; ana.estany.gestal@sergas.es (A.E.-G.); jorge.alcantara.espinosa@hotmail.com (J.M.A.-E.)

**Keywords:** quality of life, patient-reported outcome measures, age-related macular degeneration, treat and extend, anti-VEGF

## Abstract

Our objective was to evaluate changes in patient-reported outcome measures using the NEI-VFQ 25 questionnaire during a treat and extend regimen in naive neovascular Age-Related Macular Degeneration patients, and its correlation with anatomical and functional data. We conducted a prospective observational study. Patients underwent a treat and extend regimen with intravitreal ranibizumab for neovascular Age-Related Macular Degeneration. Initial response was evaluated at 4th month, and subsequently in every follow-up visit. If a clinical response was achieved, the injection interval was extended in two-week increments, up to a maximum of 12 weeks. Quality of life was assessed using the NEI-VFQ 25 questionnaire at baseline, 4th months, and 12th months. Patients were categorized as good or poor responders based on Best corrected visual acuity, central foveal thickness, intraretinal fluid, or subretinal fluid. Treatment with ranibizumab led to a significant improvement in quality of life, with a mean increase in NEI-VFQ 25 score of 4.27 points in the 12th month. No significant differences in improvement were observed between good and poor responders. Quality of life scores in neovascular Age-Related Macular Degeneration patients improved with intravitreal treatment regardless of the clinical response. The early response following the loading phase could indicate better quality of life after one year of treatment, with Best corrected visual acuity being the clinical parameter with the greatest influence on quality of life.

## 1. Introduction

Age-related macular degeneration (AMD) is the most common cause of irreversible visual impairment among people over 65 years of age worldwide [1,2], and its incidence and prevalence are expected to increase as the population ages. The neovascular form of AMD (nAMD) is characterized by the growth of a network of new blood vessels under or within the macula leading to progressive severe vision loss [3,4]. Vision loss due to nAMD results in reduced quality of life and limits activities such as mobility, facial recognition, food preparation, shopping, cleaning, watching television, reading, driving, and in some cases limiting self-care [5].

Since 2006, the use of anti-vascular endothelial growth factor (VEGF) has significantly reduced the prevalence of blindness and visual impairment due to nAMD [3,4,5,6,7]. However, anti-VEGF therapy requires multiple intravitreal injections in a treatment regimen that may last several years. Therefore, although physicians may observe improvements in vision, it is important to consider the patient’s perceived quality of life (QoL), which includes factors such as expectations, relationships, daily life, health, and disability [6,7,8,9,10].

The U.S. Food and Drug Administration (FDA) and the European Medicines Agency (EMA) recommend that researchers include patient-reported outcome measures (PROMs) when assessing the benefits of a new drug or treatment for regulatory purposes. Generic PROMs are well suited for comparisons between diseases and population, whereas disease-specific PROMs generally have higher sensitivity for disease-related characteristics One of the most commonly used vision-specific PROMS is the National Eye Institute Visual Function Questionnaire 25 (NEI-VFQ 25), which is designed to assess visual function in daily life [11]. NEI-VFQ 25 measures important areas of vision-related function in patients with chronic eye disease. It measures the extent to which vision problems affect patients’ daily functioning and self-reported health status, including factors such as social functioning and emotional well-being [12,13]. Multiple studies have confirmed the validity and reliability of this questionnaire for the treatment of nAMD [12,14,15,16,17].

Linking patient perceptions to anatomical and functional markers is a key factor in improving the quality of care and could lead to better compliance with treatment. The purpose of this study was to prospectively evaluate changes in PROMs using the NEI-VFQ 25 questionnaire during a treat and extend (T&E) treatment in naive nAMD patients, and to assess correlation with clinician-assessed anatomical and functional data.

## 2. Results

### 2.1. Clinical Measures and Response Treatment Evolution

We included 37 patients (62% female), with a mean age of 79.19 (±8.01) years, (Table 1). The total NEI-VFQ 25 score at baseline was 82.14 (±8.32) (Table 1). The classification of Macular Neovascularization (MNV) was as follows: 38% MNV type 1, 10% MNV type 2, 30% MNV type 3, and 22% mixed type. Differences between responders and non-responders were reflected in the number of injections given and their spacing throughout the year of the study. The average number of intravitreal injections was 9 (8–10), and the average treatment interval was 8 weeks (6–9).

When analyzing early clinical response at 4 months, we found that 65% of our cases presented a complete response to treatment (gain of ≥5 ETDRS letters and disappearance of all fluid), and 35% had a poor response. Patients with better response presented with intraretinal fluid (IRF) more frequently at baseline (*p* = 0.013), less subretinal fluid (SRF) (*p* = 0.002), and less sub-RPE fluid (*p* = 0.047) at the 4th month. No other significant differences were found (Table 2).

After 12 months we found that 46% of patients had a good response and 54% had a poor response. There was no significant difference in the number of patients that presented good responses at the 4th and 12th months. BCVA increased in both responders and poor responders without a significant difference (*p* = 0.292), CFT decreased in both groups, as well as the presence of IRF, SRF, and sub-RPE fluid. This indicated improvements in the clinical elements measured. The occurrence of foveal fibrosis was similar in both groups with anti-VEGF treatment (Table 3).

We found no significant differences in, BCVA, CFT, SRF, sub-RPE fluid, or development of foveal fibrosis neither at baseline nor after the loading phase (4th month) or at 12 months between those who would obtain a good or a poor response after treatment with intravitreal ranibizumab (Table 3). When analyzing the response at 12 months, we only found a significant difference in the presence of IRF at 12 months (*p* = 0.016) (Table 3).

### 2.2. Patient-Report Outcomes

**Figure 1:**
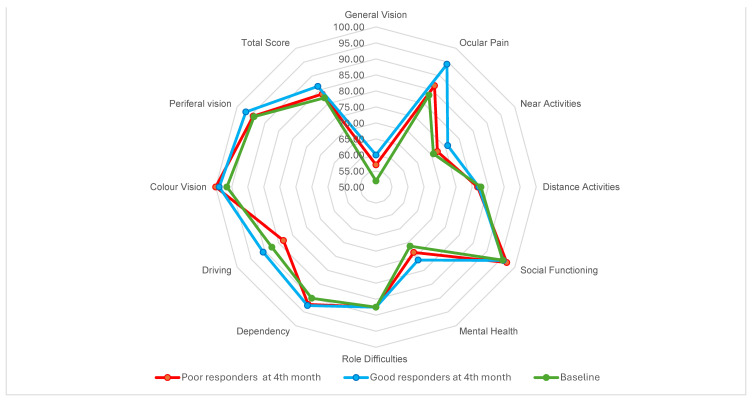
Star graph showing median NEI-VFQ 25 values from baseline to 4th month, comparing early responders to early poor responders. After the loading phase, we found that good responders presented higher average scores in several subfields. In ocular pain, good responders achieved a mean score of 94.75 (87.5–100), whereas poor responders achieved 87.5 (75–100). In near-distance activities, good responders averaged 75.85 (62.50–91.60) and poor responders 67 (54.17–89.55). In mental health good responders present a higher score 77.25 (67.19–81.25), and poor responders 75 (62.50–84.38). However, none of the differences were significant.

The total NEI-VFQ 25 score after the loading phase was 85.30 (±7.26) with no significant differences between good and poor responders. However, those who were good responders at 4 months, had significantly better total scores at the 12-month assessment (*p* = 0.048) (Figure 1).

When evaluating differences in subscores between early responders and non-responders (Figure 1) we found that the largest differences at 4 months occurred in the fields of ocular pain, near activities, and mental health. The responders had a higher score, although the differences are not significant. Those who present a good response at 4 months also present higher scores in the same fields of ocular pain, near activities, and mental health, as well as role difficulties and total score at the 12th month evaluation.

**Figure 2:**
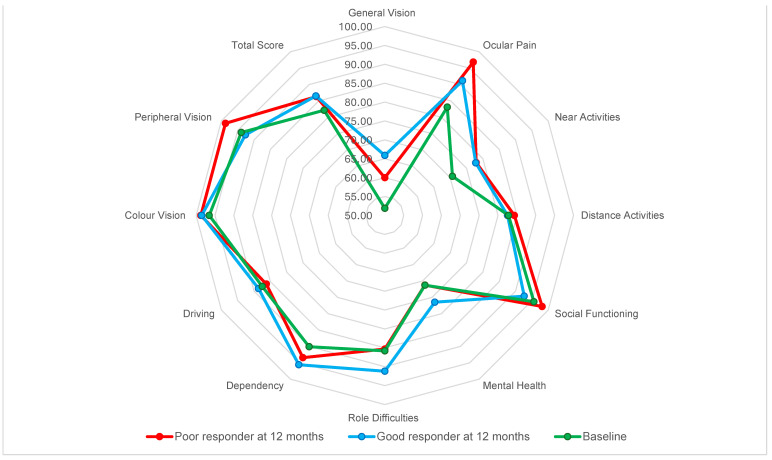
Star graph showing mean NEI-VFQ 25 values at 12th month compared to baseline in both good responders and poor responders at 12th month. None of the differences were statistically significative.

Total NEI-VFQ 25 at 12 months was 86.41 (±6.51) with no significant difference between good and poor responders. When comparing responses on each element of the NEI-VFQ 25 at 12 months, we found no significant differences between responders and non-responders. However, a trend for distance activities can be noted, with non-responders having a higher but non-significant mean value (*p* = 0.599) (Figure 2). 

In our cohort, we found that the anti-VEGF treatment significantly improved QoL with a mean difference in the total score of 4.27 (2.90–5.74) (*p* < 0.001), but the largest increase in NEI-VFQ 25 total score appeared to occur during the loading phase because the increment at month 4 was 3.17 (1.90–4.43) (*p* < 0.001). 

When analyzing the difference between NEI-VFQ 25 scores at month 4 and the scores at month 12, good responders showed mean gains of more than 5 points only in the General Vision and Distance activities categories (Table 4) although there were no significant differences between good and poor responders.

### 2.3. Relationship between Patient-Reported Outcome Measures and Clinical Measures

In our patients, we found that BCVA was the clinical parameter that had the greatest impact on PROMs.

**Figure 3:**
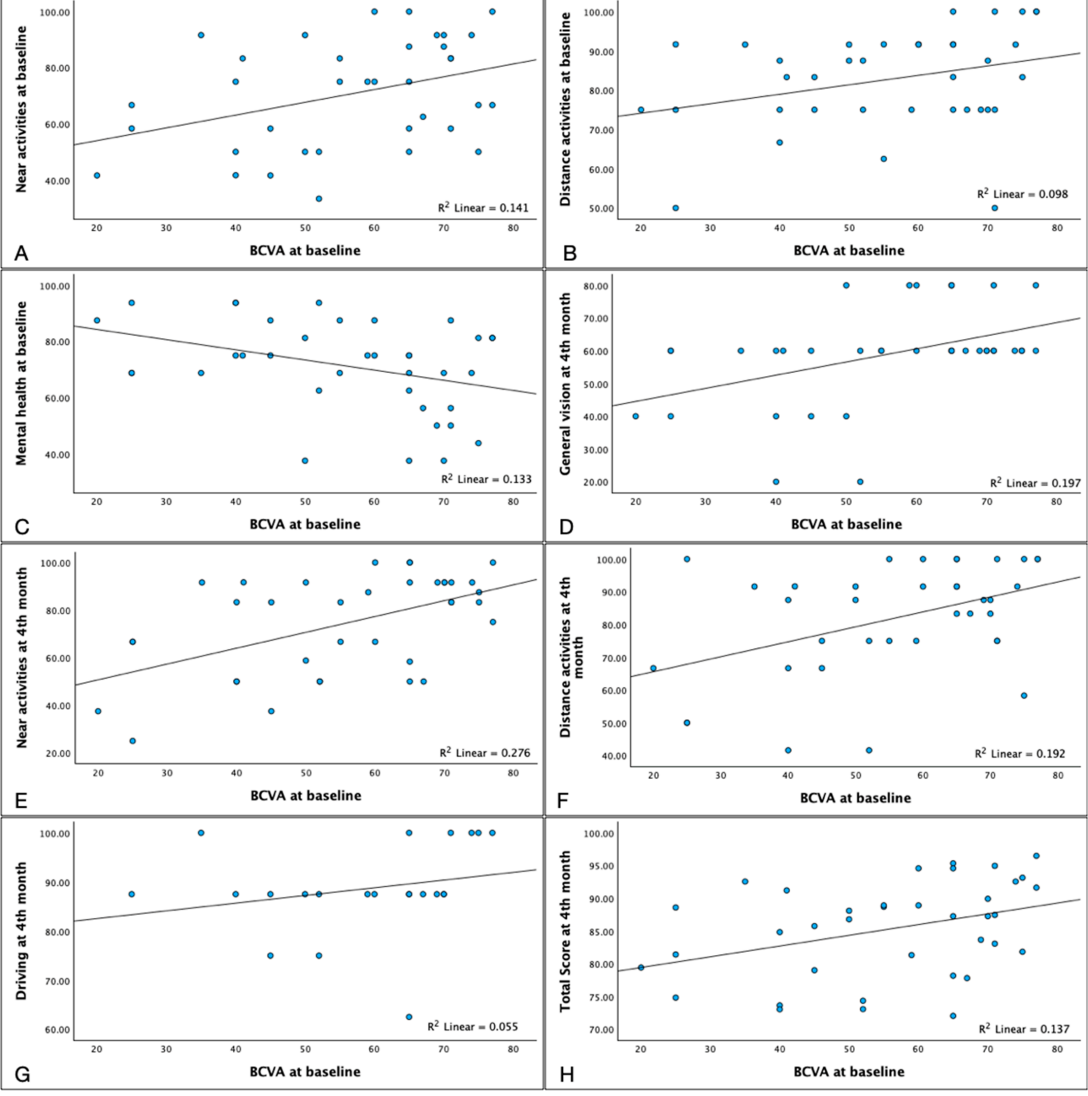
Relationship between Best Corrected Visual Acuity (BCVA) at baseline and NEI-VFQ 25 values. The BCVA achieved at baseline, positively correlated with NEI-VFQ 25 scores in the subfields of: Near activities ((**A**) Spearman’s ρ = 0.352 *p* = 0.035) Distance Activities ((**B**) ρ = −0.327 *p* = 0.048) and negatively correlated with mental health ((**C**) ρ = −0.331 *p* = 0.046). Furthermore, BCVA at baseline correlated, as well, with NEI-VFQ 25 scores at 4th months in the sections of General Vision ((**D**) ρ = 0.439 *p* = 0.007), Near activities ((**E**) ρ = 0.442 *p* = 0.006) Distance Activities ((**F**) ρ = 0.377 *p* = 0.021), Driving ((**G**) ρ = 0.431 *p* = 0.045) and Total Score ((**H**) ρ = 0.402; *p* = 0.014).

Baseline BCVA was positively correlated with baseline near activity (*p* = 0.035) and distance activity (*p* = 0.048) scores. However, it was negatively associated with baseline mental health (*p* = 0.046). We also found that baseline BCVA was positively correlated, to 4th-month scores for near activities (*p* = 0.006), distance activities (*p* = 0.021), general vision (*p* = 0.007), driving (*p* = 0.045), and the total score (*p* = 0.014) (Figure 3 and Supplementary Material, Table S1). 

**Figure 4:**
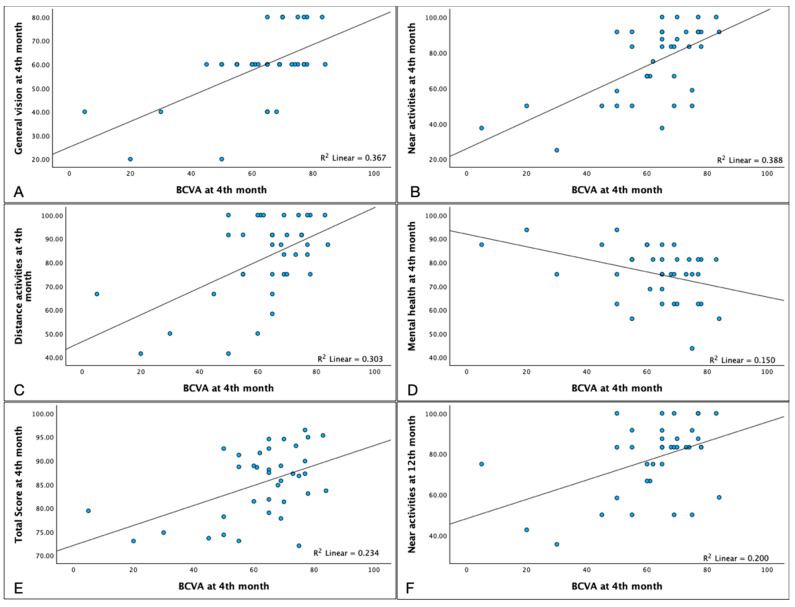
Relationship of Best Corrected Visual Acuity (BCVA) and NEI-VFQ 25 subscales at 4th month. In our series, the BCVA at baseline was positively correlated with NEI-VFQ 25 scores in the subfields of: General Vision ((**A**) Spearman’s ρ = 0.557 *p* = <0.001), Near Activities ((**B**) ρ = 0.540 *p* = 0.001), Distance Activities ((**C**) ρ = 0.378 *p* = 0.021) and Total Score ((**E**) ρ = 0.392 *p* = 0.016). BCVA at the 4th month was negatively correlated with Mental Health ((**D**) ρ = −0.367 *p* = 0.026). BCVA correlated with the Near Activities score at 12th month ((**F**) ρ = 0.419 *p* = 0.001).

When analyzing the data at month 4, BCVA at month 4 was positively correlated with general vision (*p* < 0.001), near activities (*p* = 0.001), distance activities (*p* = 0.021), and the total score (*p* = 0.016), as well as near activities at 12th month (*p* = 0.01). On the other hand, it negatively correlated with mental health (*p* = 0.026) (Figure 4, Supplementary Material Table S2). 

**Figure 5:**
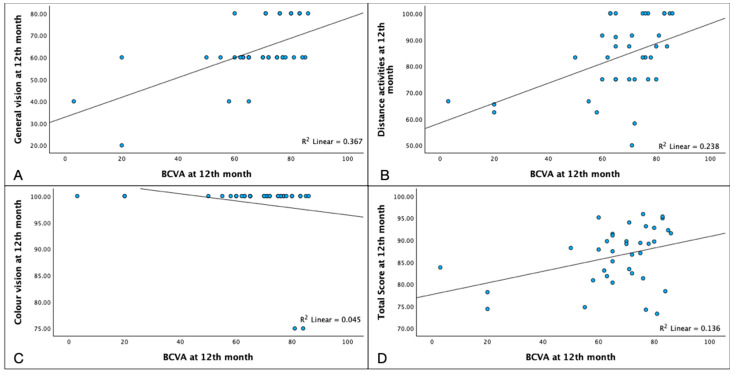
Correlations of Best Corrected Visual Acuity (BCVA) and NEI-VFQ 25 subscales at 12th month. BCVA at the 12th month was correlated with the NEI-VFQ 25 subfields of: General Vision ((**A**) Spearman’s ρ = 0.528 *p* = 0.001), Distance Activities ((**B**) ρ = 0.467 *p* = 0.004), Color Vision ((**C**) ρ = −0.325 *p* = 0.05), and Total Score ((**D**) ρ = 0.340 *p* = 0.039).

At 12 months, BCVA was positively correlated with general vision (*p* = 0.001), distance activities (*p* = 0.004), the total score (*p* = 0.039), and negatively correlated with color vision (*p* = 0.005) (Figure 5, See Supplementary Material Table S3). 

**Figure 6:**
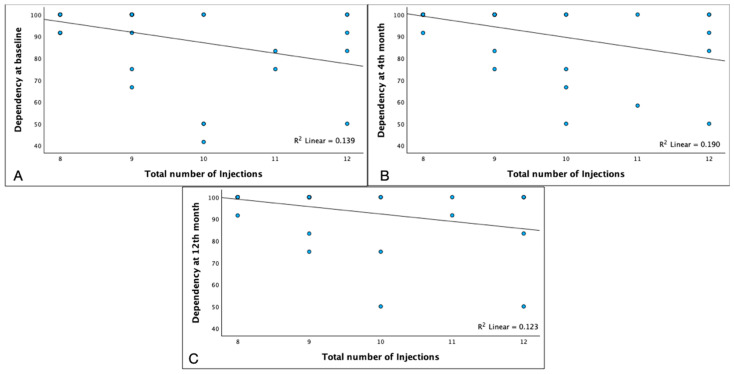
Correlations of total number of injections and NEI-VFQ 25 values for dependency at baseline 4th month and 12th month. Dependency scores in the NEI-VFQ 25 test showed a negative correlation to the total number of injections at different moments: at baseline ((**A**) Spearman’s ρ = −0.373 *p* = 0.023), at 4th months ((**B**) ρ = −0.449 *p* = 0.05) and at 12th months ((**C**) ρ = −0.333 *p* = 0.047).

We also found that the total number of injections was negatively correlated with the dependency score at baseline (*p* = 0.023), month 4 (*p* = 0.005), and month 12 (*p* = 0.047) (Figure 6, Supplementary Material Table S4). 

Regarding MNV characterization: 38% MNV type 1, 10% MNV type 2, 30% MNV type 3, and 22% mixed type (Supplementary Material, Table S5). When PROM scores were analyzed by MNV type, no differences were found in response between groups at 12 months (*p* = 0.716) (Supplementary Material, Table S6). Most patients, regardless of MNV type, improved their total NEI-VFQ 25 score with treatment (Table S6 and Figure S1 of Supplementary Material). By type (Supplementary Material, Table S6): Patients with type 1 lesions showed significant improvement only after the loading phase (*p* = 0.008). Patients with type 2 MNV did not show any significant improvements in QoL either after the loading phase (*p* = 0.068) or after one year of treatment (*p* = 0.068). There was a significant difference in NEI-VFQ 25 total score among patients with type 3 MNV at 4 months (*p* = 0.037) and at 12 months (*p* = 0.021). The total scores of mixed forms on MNV were significantly different from the baseline only at month 4 (*p* = 0.017).

## 3. Discussion

Understanding the burden of nAMD and how its treatment affects patients’ lives [9,18,19,20,21,22,23], and how the subjective data they provide correlates with the objective data provides a unique perspective for understanding the evolution of nAMD [24,25,26]. Pinelli et al. described that changes in visual function do not correlate to the anatomical changes but to biochemical alterations in both dry and nAMD [27]. PROMs are a method to improve patient management, comprehend clinical data such as BCVA and OCT fluid and reduce the risk of visual impairment [10,28]. In this study, we have prospectively evaluated the changes in quality of life during ranibizumab T&E treatment in treatment-naïve nAMD patients, using the NEI-VFQ 25 questionnaire. We correlated these changes with the assessment of anatomical and functional data.

In our series, we found that the baseline NEI-VFQ-25 composite score in our population was 82.14, which is higher than the results of previous studies (68.0–79.4) including the MARINA and ANCHOR trials [17,23,28,29,30,31]. Our results complement previous studies in other populations such as that of Oshima et al. [17]. The results showed that QoL improved linked to baseline visual acuity. In our study, baseline BCVA is one of the key elements correlated with General Vision, Near activities, and Distance activities in the early response. However, it was negatively associated with mental health, which could be explained by smaller improvements and a higher risk of vision loss [9].

Our data show that treatment of nAMD significantly improves QoL regardless of clinical response and that this improvement is clinically significant with mean gains in NEI-VFQ 25 total scores exceeding the 4-point threshold established for nAMD [12,29,32,33]. Most of this improvement occurs during the loading phase, with slower gains during the extension phase. When analyzing changes in scores for each domain reported in the NEI-VFQ 25 compared to the response, we found that regardless of response status, results at month 4 showed that General Vision, Ocular Pain, Near activities, and Mental Health scores improved. However, in the 12-month assessment, only those who present a good response present changes greater than 5 points at General Vision and Near activities.

The long-term benefits of treatment did not differ between responders and poor responders, as both groups experienced similar improvements in QoL scores. This could indicate that, although it is not possible to achieve a complete anatomical response in all the patients, treatment provides improvements in the QoL due to stabilization of the VA, reduction in central foveal thickness, and reduction in the presence of fluid in all the anatomical compartments [10,12,27,34]. Furthermore, we noticed an interesting difference: patients who showed a good early response after the loading phase recorded significantly higher QoL scores at 12 months and showed a tendency to gain improvements in areas such as General vision and Near activities during the extension phase, which may indicate that improvements achieved at short term response affect long term QoL. 

Incorporating PROMs into the evaluation of clinical markers provides new perspectives for understanding treatment response [29]. Although we found significant results at 12 months, they were only found in the presence of IRF, as one of the main factors characterizing exudative activity. Reducing exudative activity resulted in a better NEI-VFQ 25 score, even when some fluid persists. The lack of significant associations between other clinical markers and the response can be attributed to the multifactorial pathophysiology of nAMD and requires further investigation.

When considering MNV types, we found that the frequency of different MNV types was similar to that reported previously [35]. In most of our patients, significant differences in QoL scores occurred after the loading phase and improvements were maintained during the extension phase until month 12, although no significant improvement in QoL was observed during this period. In our study, patients with type 3 MNV reported lower QoL. This may be attributed to the fact that type 3 MNV is often associated with areas of atrophy which may lead to further deterioration of vision [4,36,37,38,39,40,41] and tends to affect both eyes [35,39]. Ranibizumab treatment provided significant changes during the extension phase of treatment, and similar improvements in total QoL scores were achieved compared with other MNV types. 

This study presents several limitations: First, the definition of clinical response, where no fluid was tolerated, regardless of its location, and a minimum five-letter improvement was required to be considered a good response. Another limitation is the small sample size, originating from a single center. Another limitation is that we did not consider data from the fellow eye in our analysis. On the other hand, our results are limited to ranibizumab and a T&E regimen with two-week increment intervals, as T&E is considered the most used treatment regimen in clinical practice [5,6,7]. Future studies are required to evaluate other anti-VEGF agents that allow longer intervals of extension, such as aflibercept [30], brolucizumab, or faricimab as it is necessary to consider anatomical and biochemical data as well as functional data and patients’ perceptions to obtain optimal results in management of nAMD [7,10,27,28,42].

## 4. Materials and Design

### 4.1. Patient Selection Criteria

Inclusion criteria for all study patients were defined as neovascularization secondary to AMD, older than 55 years, VA greater than 25 letters at baseline, and no history of prior anti-VEGF therapy (nAMD-naïve). Exclusion criteria included any retinal disease other than nAMD that could explain the presence of fluid. Additionally, patients who had undergone previous retinal surgery or had signs of intraocular inflammation were excluded. 

All enrolled subjects were nAMD-naïve and received a T&E regimen of intravitreal injections: three monthly injections with ranibizumab (Lucentis^®^ Novartis, Basel, Switzerland) with follow-up examinations one month after the third injection, to determine early treatment response (4 months evaluation). After the loading phase, a new intravitreal injection of ranibizumab and an evaluation of the response were performed in every visit. If complete response was achieved the next injection was postponed two weeks, up to 12 weeks; if partial response was achieved, the interval between ranibizumab injections was maintained; if a deterioration of visual acuity (VA) or increased exudative activity was detected, the interval between injections was reduced to a minimum of 4 weeks.

### 4.2. Clinical Measures

All patients were evaluated to determine the best corrected visual acuity (BCVA). In addition, ophthalmoscopy, and optical coherence tomography (OCT) were performed on every visit under pharmacological mydriasis (Spectralis^®^; Heidelberg Engineering, Heidelberg, Germany, and SS-OCT-DRI-TRITON^®^, Topcon Corp Inc., Tokyo, Japan). All patients in this study were imaged using 3 × 3 mm, 6 × 6 mm, and 9 mm scan patterns centered on the fovea. All patients underwent fluorescein angiography (HRA-2^®^; Heidelberg Engineering, Heidelberg, Germany) at baseline. 

Macular neovascularization (MNV) was classified according to the consensus nomenclature of Spaide et al. [43].

### 4.3. Classification of Clinical Response

Classification of patient response was based on Amoaku et al. [44]. Good morphological and/functional response was achieved when OCT showed an absence of intraretinal fluid (IRF), subretinal fluid (SRF), normalization of the central foveal thickness (CFT), and improvement of at least five ETDRS letters. Poor responders were defined as patients with less than 25% reduction in OCT retinal thickness from the baseline, with persistent or new IRF, SRF, or change in visual acuity inferior to five letters after anti-VEGF therapy. Non-responders were defined as those patients who showed an increase in central retinal thickness, developed subretinal fibrosis, or showed an increase in IRF or SRF. Non-responders and poor responders were pooled in a single group for analysis purposes. Response evaluation has been conducted after the loading phase (month 4) and at month 12.

### 4.4. Patient-Reported Outcome Measures

Patient-reported outcome measures were assessed using the NEI-VFQ-25 questionnaire, which covers various aspects including general health, general vision, ocular pain, near vision activities, distance vision activities, social functioning, vision-specific role difficulties, vision-specific mental health, dependence due to vision, driving, peripheral vision, and color vision [13]. Each individual’s response was converted to a score between 0 and 100, with higher scores reflecting better vision-related function. Items in the NEI-VFQ 25 questionnaire were categorized into 12 subscales by grouping related questions together and calculating each subscale score by averaging the scores for all related questions in that specific subscale. The NEI-VFQ 25 composite score was calculated by averaging the scores of all subscales [12]. The NEI-VFQ 25 questionnaire was administered at the baseline, after completing the loading phase (month 4), and at month 12 by a member of our team to account for reading difficulties [18]. The results were analyzed in accordance with the published NEI-VFQ 25 guidelines. A 4-point change in the overall NEI-VFQ 25 was considered a minimal clinically meaningful change in the NEI-VFQ 25 score of an individual [12,33].

### 4.5. Statistical Analysis

Differences between good and poor responders groups were explored using statistical tests: categorical variables were explored using Chi^2^; in continuous variables, a Shapiro–Wilk test for checking normality and a *t*-student or a Wilcoxon or Mann–Whitney test. Five of 37 patients were treated for nAMD in both of their eyes, however, for the purpose of the analysis, only right eyes were included. Data were analyzed both after a loading phase at the 4th month and at the 12th month.

## 5. Conclusions

Our study has shown that quality of life scores in nAMD improve with intravitreal treatment regardless of clinical response. However, response after the loading phase may be an indicator of improved QoL after one year of treatment, as patients with good response after the loading phase reported higher QoL scores at 12 months. 

In our patients, we found that the clinical parameter with the greatest influence on PROM was BCVA associated with short- and long-term: general vision, near activities, and distance activities. We also observed that the number of injections correlated with dependency scores and that different MNV types improved with ranibizumab treatment, despite MNV type. Future studies are needed to evaluate longer follow-up periods and different treatment agents.

## Figures and Tables

**Table 1:** Demographic data, PROMs score evolution and response characteristics in our cohort. Abbreviatures: National Eye Institute Visual Functioning Questionnaire-25 (NEI-VFQ 25), Interquartile range (IQR).

	Total n = 37	Poor Responder 12 Months n = 20 (54%)	Good Responder 12 Months n = 17 (46%)	*p*-Value
Sex	Male 14 (38%)	Male 8 (40%)	Male 6 (35%)	0.776 ^+^
	Female 23 (62%)	Female 12 (60%)	Female 11 (65%)	
Age	79.19 (±8.01)	78.85 (±7.93)	79.59 (±8.34)	0.785 ^+^
Total NEI-VFQ 25 score at baseline (Mean, SD)	82.14 (±8.32)	81.92 (±9.42)	82.39 (±7.09)	0.865 ^+^
Total NEI-VFQ 25 score at month 4 (Mean, SD)	85.30 (±7.26)	85.52 (±7.62)	85.05 (±7.05)	0.846 ^+^
Total NEI-VFQ 25 score at month 12 (Mean, SD)	86.41 (±6.51)	86.31 (±7.04)	86.52 (±6.03)	0.924 ^+^
Response at month 4				0.743 *
Poor responder (n, %)	13, 35%	8, 40%	6, 35%	
Good responder (n, %)	24, 65%	12, 60%	11, 65%	
Total injections (Mean, IQR)	9 (8–10)	9 (8–10)	9 (8–9)	0.059 ^‡^
Weeks between injection (Mean, IQR)	8 (6–9)	7 (6–8)	9 (7–9)	0.048 ^‡^

* Tested by Chi^2^, ^+^ Tested by Student-*t*, ^‡^ Non-normal distribution (Tested by Shapiro-Wilks test), variable tested by Mann-Whitney test.

**Table 2:** Clinical outcomes evolution and response at month 4. Abbreviatures: Best Corrected Visual Acuity (BCVA), Central Foveal Thickness (CFT), Intraretinal Fluid (IRF), Subretinal fluid (SRF), Sub-Retinal pigmented epithelium fluid (sub-RPE fluid), Interquartile range (IQR).

Variables	Total n = 37	Poor Responder 4 Months n = 13 (35%)	Good Responder 4 Months n = 24 (65%)	*p*-Value
BCVA at baseline (Median, IQR)	60 (43–70)	65 (45–72)	57 (42–67)	0.291 ^‡^
BCVA at month 4 (Median, IQR)	65 (55–75)	65 (53–73)	69 (56–75)	0.369 ^‡^
BCVA at month 12 (Median, IQR)	71 (63–77)	70 (61–80)	71 (63–77)	1.000 ^‡^
CFT at baseline (Median µm, IQR)	310 (267–364)	333 (234–406)	296 (266–354)	0.743 ^‡^
CFT at month 4 (Median µm, IQR)	214 (200–250)	205 (195–296)	216 (201–247)	0.888 ^‡^
CFT at month 12 (Median µm, IQR)	232 (212–274)	229 (195–319)	233 (214–261)	0.987 ^‡^
IRF at baseline (n, %)				0.013 *
Absence	13, 35%	8, 62%	5, 21%	
Present	24, 65%	5, 38%	19, 79%	
IRF at month 4 (n, %)				0.874 *
Absence	29, 78%	10, 77%	19, 79%	
Present	8, 22%	3, 23%	5, 21%	
SRF at baseline (n, %)				0.896 *
Absence	9, 24%	3, 23%	6, 25%	
Present	28, 76%	10, 77%	18, 75%	
SRF at month 4 (n, %)				0.002 *
Absence	28, 76%	6, 46%	22, 92%	
Present	9, 24%	7, 54%	2, 8%	
SRF at month 12 (n, %)				0.320 *
Absence	29,78%	9, 69%	20, 83%	
Present	8, 22%	4, 31%	4, 17%	
Sub-RPE fluid at baseline (n, %)				0.515 *
Absence	11, 30%	3, 23%	8, 33%	
Present	26, 70%	10, 77%	16, 67%	
Sub-RPE fluid at month 4 (n, %)				0.047 *
Absence	26, 70%	6, 46%	20, 83%	
Present	11, 30%	7, 54%	4, 17%	
Sub-RPE fluid at month 12 (n, %)				0.405
Absence	31, 84%	10, 77%	21, 88%	
Present	6, 76%	3, 23%	3, 12%	
Fibrosis at baseline (n, %)				1.000
Absence	37, 100%	37, 100%	37, 100%	
Present	0, 0%	0, 0%	0, 0%	
Fibrosis at month 4 (n, %)				0.806 *
Absence	32, 86%	11, 85%	21, 88%	
Present	5, 14%	2, 15%	3, 12%	
Fibrosis at month 12 (n, %)				0.686 *
Absence	30, 81%	11, 85%	19, 79%	
Present	7, 19%	2, 15%	5, 21%	

* Tested by Chi^2^, ^‡^ Non-normal distribution (tested by Shapiro–Wilk test), variable tested by Mann–Whitney test.

**Table 3:** Clinical outcomes evolution and response at month 12. Abbreviatures: Best Corrected Visual Acuity (BCVA), Central Foveal Thickness (CFT), Intraretinal Fluid (IRF), Subretinal fluid (SRF), Sub-Retinal pigmented epithelium fluid (sub-RPE fluid), Interquartile range (IQR).

Variables	Total n = 37	Poor Responder 12 Months n = 20 (54%)	Good Responder 12 Months n = 17 (46%)	*p*-Value
BCVA at baseline (median, IQR)	60 (43–70)	58 (41–65)	65 (45–71)	0.292 *
BCVA at month 4 (Median, IQR)	65 (55–75)	65 (55–69)	69 (60–77)	0.264 ^‡^
BCVA at month 12 (Median, IQR)	71 (63–77)	68 (62–76)	72 (65–80)	0.306 ^‡^
CFT at baseline (Median µm, IQR)	310 (267–364)	341 (256–372)	290 (270–345)	0.503 ^‡^
CFT at month 4 (Median µm, IQR)	214 (200–250)	220 (193–249)	211 (203–250)	0.737 ^‡^
CFT at months 12 (Median µm, IQR)	232 (212–274)	235 (223–261)	224 (205–275)	0.503 ^‡^
IRF at baseline (n, %)				0.501 *
Absence	13, 35%	8, 40%	5, 29%	
Present	24, 65%	12, 60%	12, 71%	
IRF at month 4 (n, %)				0.179 *
Absence	29, 78%	14, 70%	15, 88%	
Present	8, 22%	6, 30%	2, 12%	
IRF at month 12 (n, %)				0.016 *
Absence	28, 76%	12, 60%	16, 94%	
Present	9, 24%	8, 40%	1, 6%	
SRF at baseline (n, %)				0.383 *
Absence	9, 24%	6, 30%	3, 18%	
Present	28, 76%	14, 70%	14, 82%	
SRF at month 4 (n, %)				0.917 *
Absence	28, 76%	15, 75%	13, 76	
Present	9, 24%	5, 25%	4, 24%	
SRF at month 12 (n, %)				0.179 *
Absence	29, 78%	14, 70%	15, 88%	
Present	8, 22%	6, 40%	2, 12%	
Sub-RPE fluid at baseline (n, %)				0.160 *
Absence	11, 30%	4, 20%	7, 41%	
Present	26, 70%	16, 80%	10, 59%	
Sub-RPE fluid at month 4 (n,%)				0.138 *
Absence	26, 70%	12, 60%	14, 82%	
Present	11, 30%	8, 40%	3, 18%	
Sub-RPE fluid at month 4 (n,%)				0.498 *
Absence	31, 84%	16, 80%	15, 88%	
Present	6, 76%	4, 20%	2, 12%	
Fibrosis at baseline (n,%)				0.622 *
Absence	37, 100%	20, 100%	17, 100%	
Present	0, 0%	0, 0%	0, 0%	
Fibrosis at month 4 (n,%)				0.211 *
Absence	32, 86%	16, 80%	16, 94%	
Present	5, 14%	4, 20%	1, 6%	
Fibrosis at month 4 (n,%)				0.856 *
Absence	30, 81%	16, 80%	14, 82%	
Present	7, 19%	4, 20%	3, 18%	

* Tested by Chi^2^, ^‡^ Non-normal distribution (tested by Shapiro–Wilk test), variable tested by Mann–Whitney test.

**Table 4:** Change in NEI-VFQ 25 values from 4th month to 12th month and response at 12th months.

Variables	Total n = 37	Poor Responder 12 Months n = 20 (54%)	Good Responder 12 Months n = 17 (46%)	*p*-Value
General Vision Mean, (SD)	3.78 (±12.33)	1.00 (±10.21)	7.06 (±14.04)	0.244 *
Ocular Pain Mean, (SD)	2.70 (±8.40)	4.38 (±7.34)	0.74 (±9.34)	0.341 *
Near Activities Mean, (SD)	3.35 (±15.20)	4.61 (±13.74)	1.87 (±17.06)	0.460 *
Distance Activities Mean, (SD)	1.54 (±1.38)	−1.90 (±9.68)	5.57 (±12.17)	0.341 *
Social Functioning Mean, (SD)	−0.68 (±5.06)	0.00 (±4.10)	−1.47 (±6.06)	0.577 *
Mental Health Mean, (SD)	−1.69 (±8.54)	−3.12 (±9.62)	0.00 (±6.99)	0.357 *
Role Difficulties Mean, (SD)	2.21 (±9.58)	2.83 (±7.23)	1.47 (±11.59)	0.357 *
Dependency Mean, (SD)	1.39 (±5.81)	0.88 (±2.63)	1.96 (±8.09)	0.851 *
Driving Mean, (SD)	−1.19 (±3.76)	−1.25 (±3.95)	−1.14 (±3.77)	0.973 *
Color Vision Mean, (SD)	−0.69 (±4.17)	0.00 (±0.00)	−1.56 (±6.25)	0.765 *
Peripheral Vision Mean, (SD)	0.00 (±5.89)	0.00 (±0.00)	0.00 (±8.84)	1.000 *
Total Score Mean, (SD)	1.11 (±3.66)	0.80 (±3.07)	1.47 (±4.33)	0.775 *

* Non-normal distribution (tested by Shapiro–Wilk test), variable tested by Mann–Whitney test.

## Data Availability

The data presented in this study are available on request from the corresponding author.

## Supplementary Materials

The following supporting information can be downloaded at: https://www.mdpi.com/article/10.3390/ph17020157/s1, Supplementary Table S1: Correlations between baseline BCVA and QoL, Supplementary Table S2: Correlations between BCVA at month 4 and QoL, Supplementary Table S3: Correlations between BCVA at month 12 and QoL, Supplementary Table S4: Correlations between number of injections and QoL. Supplementary Table S5: MNV type and response, Supplementary Table S6: MNV type and NEI-VFQ 25 values, Supplementary Table S7: Response at month 4 and NEI-VFQ 25 scores at month 12 and Supplementary Figure S1: Evolution of Median of QoL Scores and MNV type.

## Abbreviations

AMD	Age-Related Macular Degeneration.
EMA	European Medicines Agency
ETDRS	Early Treatment Diabetic Retinopathy Study
FDA	Food And Drug Administration
IRF	Intraretinal Fluid
MNV	Macular Neovascularization
nAMD	Neovascular Age-Related Macular Degeneration
NEI-VFQ 25	National Eye Institute Visual Functioning Questionnaire–25
OCT	Optical Coherence Tomography
PROMs	Patient Reported Outcome Measure
QoL	Quality of Life
RPE	Retinal Pigmented Epithelium
SRF	Subretinal Fluid
T&E	Treat and Extend
BCVA	Best Corrected Visual Acuity
VEGF	Vascular Endothelial Growth Factor
