# Supplementary material for: Relationships between Patient-Reported Outcome Measures and Clinical Measures in Naïve Neovascular Age-Related Macular Degeneration Patients Treated with Intravitreal Ranibizumab
Source: Pharmaceuticals (Basel). 2024 Jan 25;17(2):157. doi: 10.3390/ph17020157 (PMC10893278; doi:10.3390/ph17020157)
Supplement: Supplementary file 1 [file pharmaceuticals-17-00157-s001.zip › pharmaceuticals-2792375-supplementary.pdf]

pharmaceuticals

Supplementary material

7.1 Supplementary Table S1: Correlations between baseline BCVA and QoL

	BCVA at baseline	
	Correlation Coefficient	p-Value
General vision at baseline	0.166	0.327
Ocular pain at baseline	0.133	0.432
Near activities at baseline	0.352	0.035
Distance activities at baseline	0.327	0.048
Social functioning at baseline	- 0.041	0.81
Mental health at baseline	- 0.331	0.046
Role difficulties at baseline	0.213	0.206
Dependency at baseline	0.119	0.481
Driving at baseline	0.405	0.061
Colour vision at baseline	0.034	0.844
Peripheral vision at baseline	0.118	0.487
General vision at 4th month	0.439	0.007
Ocular pain at 4th month	0.122	0.473
Near activities at 4th month	0.442	0.006
Distance activities at 4th month	0.377	0.021
Social functioning at 4th month	0.213	0.206
Mental health at 4th month	- 0.258	0.123
Role difficulties at 4th month	0.148	0.383
Dependency at 4th month	0.190	0.259
Driving at 4th month	0.431	0.045
Colour vision at 4th month	- 0.057	0.741
Peripheral vision at 4th month	0.246	0.142
General vision at 12th month	0.139	0.41
Ocular pain at 12th month	0.015	0.931
Near activities at 12th month	0.209	0.215
Distance activities at 12th month	0.255	0.128
Social functioning at 12th month	- 0.073	0.67
Mental health at 12th month	- 0.117	0.492
Role difficulties at 12th month	0.078	0.645
Dependency at 12th month	0.173	0.312
Driving at 12th month	0.327	0.148
Colour vision at 12th month	-0.135	0.427
Peripheral vision at 12th month	0.145	0.391
Total score at baseline	0.239	0.154

Total score at 4th month	0.402	0.014
Total score at 12th month	0.192	0.256

Supplementary Table I Correlations between baseline BCVA and QoL (Spearman's Rho)

7.2 Supplementary Table S2: Correlations between BCVA at 4th Month and QoL

	**BCVA at 4th month**	
	Correlation Coefficient	p-Value
**General vision at 4th month**	**0.557**	**< 0.0001**
Ocular pain at 4th month	- 0.037	0.829
**Near activities at 4th month**	**0.540**	**0.001**
**Distance activities at 4th month**	**0.378**	**0.021**
Social functioning at 4th month	0.128	0.45
**Mental health at 4th month**	**- 0.367**	**0.026**
Role difficulties at 4th month	0.190	0.26
Dependency at 4th month	0.164	0.331
Driving at 4th month	0.405	0.061
Colour vision at 4th month	- 0.163	0.341
Peripheral vision at 4th month	0.282	0.091
General vision at 12th month	0.384	0.019
Ocular pain at 12th month	0.044	0.795
**Near activities at 12th month**	**0.419**	**0.01**
Distance activities at 12th month	0.281	0.092
Social functioning at 12th month	- 0.133	0.432
Mental health at 12th month	- 0.138	0.414
Role difficulties at 12th month	0.163	0.336
Dependency at 12th month	0.196	0.253
Driving at 12th month	0.253	0.268
Colour vision at 12th month	-0.32	0.053
Peripheral vision at 12th month	0.094	0.578
**Total score at 4th month**	**0.392**	**0.016**
Total score at 12th month	0.277	0.097

Supplementary Table II Correlations between BCVA at 4th Month and QoL. (Spearman's Rho)

7.3 Supplementary Table S3 Correlations between BCVA at 12th month and QoL

	BCVA at 12th month	
	Correlation Coefficient	p-Value
**General vision at 12th month**	**0.528**	**0.001**
Ocular pain at 12th month	0.215	0.202
Near activities at 12th month	0.187	0.268
**Distance activities at 12th month**	**0.467**	**0.004**
Social functioning at 12th month	0.076	0.654
Mental health at 12th month	- 0.022	0.896
Role difficulties at 12th month	0.196	0.245
Dependency at 12th month	0.082	0.634
Driving at 12th month	0.183	0.428
**Colour vision at 12th month**	**- 0.325**	**0.05**
Peripheral vision at 12th month	0.183	0.277
**Total score at 12th month**	**0.340**	**0.039**

Supplementary Table III Correlations between BCVA at 4th Month and QoL (Spearman's Rho)

7.4 Supplementary Table S4 Correlations between number of injections and QoL (Spearman's Rho)

	Number of Injections	
	Correlation Coefficient	p-Value
General vision at baseline	0.172	0.308
Ocular pain at baseline	- 0.023	0.892
Near activities at baseline	- 0.128	0.458
Distance activities at baseline	0.078	0.648
Social functioning at baseline	- 0.142	0.403
Mental health at baseline	- 0.169	0.318
Role difficulties at baseline	- 0.028	0.869
**Dependency at baseline**	**- 0.373**	**0.023**
Driving at baseline	- 0.100	0.659
Colour vision at baseline	- 0.028	0.869
Peripheral vision at baseline	0.067	0.691
General vision at 4th month	0.146	0.389
Ocular pain at 4th month	0.089	0.601
Near activities at 4th month	- 0.076	0.654
Distance activities at 4th month	0.148	0.383
Social functioning at 4th month	- 0.184	0.274
Mental health at 4th month	- 0.276	0.098

Role difficulties at 4th month	- 0.128	0.45
**Dependency at 4th month**	**- 0.449**	**0.005**
Driving at 4th month	- 0.203	0.364
Colour vision at 4th month	- 0.266	0.117
Peripheral vision at 4th month	0.081	0.632
General vision at 12th month	- 0.009	0.958
Ocular pain at 12th month	0.226	0.179
Near activities at 12th month	- 0.275	0.099
Distance activities at 12th month	0.103	0.546
Social functioning at 12th month	0.119	0.484
Mental health at 12th month	- 0.095	0.575
Role difficulties at 12th month	- 0.121	0.476
**Dependency at 12th month**	**- 0.333**	**0.047**
**Driving at 12th month**	- 0.156	0.5
**Colour vision at 12th month**	- 0.176	0.297
**Peripheral vision at 12th month**	0.081	0.632
**Total score at baseline**	- 0.168	0.321
**Total score at 4th month**	- 0.199	0.239
**Total score at 12th month**	- 0.149	0.379

Supplementary Table VI Correlations between number of injections and QoL (Spearman's Rho)

7.5 Supplementary Table S5: MNV type and response

**MNV Type**	**Total N= 37**	**Poor responders at 12th month N= 20 (54%)**	**Good responders at 12th month N= 17 (46%)**	**p-value**
				0,7157[1]
**Type 1**	14, 37.8%	7, 35%	7, 41.2%	
**Type 2**	4, 10.8%	3, 15%	1, 5.9%	
**Type 3**	11, 29.7%	5, 25%	6, 35.3%	
**Type 4**	8, 21.7%	5, 25%	3, 17.6%	

[1] Tested by Chi²

Supplementary Table V MNV type and response

7.6 Supplementary Table S6: MNV type and NEI-VFQ 25 values

		Mean	SD	Median	
Type 1	Total Score at Baseline	83.19	8.27	83.93	
	Total Score 4th month	86.51	7.04	87.36	Change from baseline to 4th month: 2.67 ($p = 0.008$)
	Total Score 12th Month	87.54	6.61	89.411	Change from 4th month to 12th Month: 1.26 ($p = 0.209$)
					Change from baseline to 12th Month: 3.11 ($p=0.002$)
Type 2	Total Score at Baseline	83.85	7.62	86.07	
	Total Score 4th month	85.93	8.39	88.76	Change from baseline to 4th month: 1.83 ($p = 0.068$)
	Total Score 12th Month	87.10	8.68	89.24	Change from 4th month to 12th Month: 1.83 ($p = 0.068$)
					Change from baseline to 12th Month: 1.83 ($p = 0.068$)
Type 3	Total Score Baseline	78.84	9.93	79.70	
	Total Score 4th month	82.01	8.22	79.01	Change from baseline to 4th month: 2.09 ($p = 0.037$)
	Total Score 12th Month	83.90	7.05	83.10	Change from 4th month to 12th Month: 2.18 ($p = 0.029$)
					Change from baseline to 12th Month: 2.31 ($p=0.021$)
Mixed forms	Total Score Baseline	83.96	6.24	84.39	
	Total Score 4th month	87.41	5.22	86.51	Change from baseline to 4th month: 2.38 ($p = 0.017$)
	Total Score 12th Month	87.54	4.37	87.44	Change from 4th month to 12th Month: 0.17 ($p = 0.866$)
					Change from baseline to 12th Month: 1.82 ($p=0.069$)

Contrasted using Wilcoxon

Supplementary Table VI MNV type and NEI-VFQ 25 values

7.7 Supplementary Table S7: Response at 4th month and NEI-VFQ 25 scores at 12th month

Variables	Total N= 37	Poor responders at 4th month N = 13 (35%)	Good responders at 4th month N = 24 (65%)	P-values
**Ocular pain at 12th month**	100 (87.5-100)	87.5 (87.50-100)	100 (90.63-100)	0.112 *
**Near activities at 12th month**	83.30 (66.67-87.50)	75 (54.17-85.42)	83.30 (77.01-91.65)	0.115 *
**Distance activities at 12th month**	83.33 (75-100)	87.5 (64.59-95.50)	83.31 (75-100)	0.438 *
**Social fnctioning at 12th month**	100 (87.5-100)	100 (93.75-100)	100 (87.5-100)	0.774 *

**Mental health at 12th month**	75 (68.75-81.25)	68.80 (68.75-84.38)	75 (68.75-81.25)	0.77 *
**Role difficulties at 12th month**	87.50 (75-100)	87.50 (75-100)	93.75 (87.5-100)	0.139 *
**Dependency at 12th month**	100 (100-100)	100 (91.67-100)	100 (100-100)	0.839 *
**Colour vision at 12th month**	100 (100-100)	100 (100-100)	100 (100-100)	0.655 *
**Peripheral vision at 12th month**	100 (100-100)	100 (87.50-100)	100 (100-100)	0.198 *
**Total score at 12th month**	86.41(±6.51)	83.56 (±6.86)	87.95 (±5.89)	0.048

Supplementary Table VII: Response at 4th month and NEI-VFQ 25 scores at 12th month

7.8 **Supplementary Figure S1:** Evolution of Median of QoL Scores and MNV type

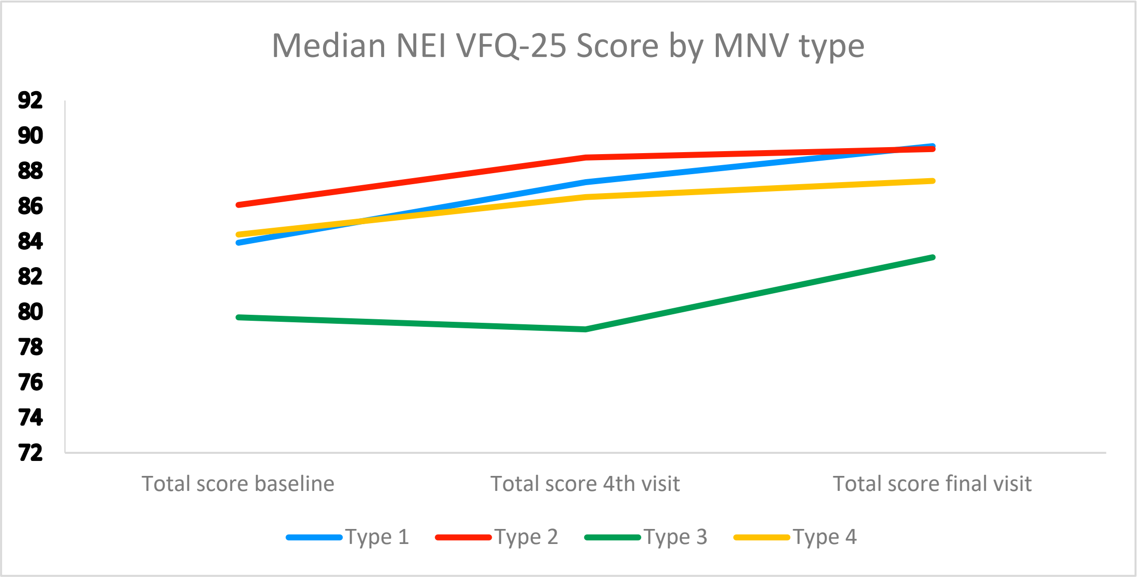

Supplementary Figure I: Evolution of Median of Qol Scores and MNV type
